# Understanding Neuromuscular Health and Disease: Advances in Genetics, Omics, and Molecular Function

**DOI:** 10.3390/jpm11050438

**Published:** 2021-05-20

**Authors:** William J. Duddy, Stephanie Duguez

**Affiliations:** Northern Ireland Center for Stratified/Personalized Medicine, Ulster University, Glenshane Road, Derry-Londonderry BT47 6SB, UK

The field of neuromuscular research has seen considerable recent advances in the molecular and cellular understanding of muscle biology, and the treatment of neuromuscular disease. These advances are at the forefront of modern molecular methodologies, often integrating across wet-lab cell and tissue models, dry-lab computational approaches, and clinical studies. The continuing development and application of multiomics methods offer particular challenges and opportunities, not least in the potential for personalized medicine. More than 500 different genes are known to be associated with neuromuscular disorders [1], and the identification of causative mutations has allowed the development of personalized therapies [1,2]. However, even if great progress has been made during the last two decades in different subgroups of neuromuscular disorders, there are still numerous challenges to resolve, such as the optimization of therapeutic knock-down strategies [3], targeting specific muscles and/or tissues of the nervous system [1], identifying genetic modifiers that can impair a therapeutic strategy [2], targeting common pathways being affected in different patient subgroups for a given disease [4,5], or understanding the impact of neuromuscular disorders on other tissues that could be affected but may be understudied.

This Special Issue, entitled “Understanding Neuromuscular Health and Disease: Advances in Genetics, Omics, and Molecular Function”, encompasses some 15 publications from colleagues working on a diverse range of neuromuscular diseases, including Duchenne muscular dystrophy [6,7,8,9], facioscapulohumeral dystrophy [3,10,11], amyotrophic lateral sclerosis [4,5,12], spinal muscular atrophy [2], Emery–Dreifuss muscular dystrophy [13], and rheumatoid arthritis [14]. Looking across diseases, several themes are recurrent, such as the efforts to identify genotype–phenotype correlations in DMD [6,7,9] and ALS [4,5], the quest for effective biomarkers in many neuromuscular conditions [2,8,10,14], and the use of genomic and multi-omic approaches towards better ways to identify biomarkers and to understand disease [10,12,13].

The search for genotype–phenotype correlations can be aimed at the improved understanding of disease [4,5,7,9], but may also be relevant to potential therapeutic outcomes [6]. Of relevance to this Special Issue are genotype–phenotype correlations in DMD [6,7,9] and in ALS [4,5]. It is interesting to contrast the state of these investigations in these two conditions, differences which are related to the underlying genetics: DMD being due to mutations at a single gene, and therefore correlations being sought between clinical outcomes and specific mutation patterns within that gene [6,7,9]; ALS being a disease of unclear aetiology for the majority of patients and the focus of these genotype–phenotype investigations thus being on the relationship of different genes and their functional roles to the implicated mechanisms [4] or clinical outcomes of the condition [5].

The use of genomics and multi-omics approaches is a theme which itself cuts across the aims of current research, from the overlay of multiple omics data to achieve a global perspective and new understanding of Emery–Dreifuss muscular dystrophy [13], to the identification of novel circulating miRNA and protein biomarkers for FSHD using multi-omics [10], through to the application of machine learning to the genomics of ALS, which is aimed at understanding the molecular basis of this disease (and is also relevant to genotype–phenotype correlations) [12]. Deciphering the pathways and gene mutations involved in neuromuscular diseases may allow for the development of computational models helping our understanding of muscle pathologies, which could enable preclinical studies of neuromuscular diseases in the context of personalized medicine [15].

Aside from therapeutic strategy development, the use of biomarkers may be critical as disease trackers for the development of effective therapeutics (for example, in FSHD [10]), but also to the personalized tailoring of existing treatments (in DMD [8], and in rheumatoid arthritis [14]), and may prove useful in a broad sense for improved stratification, diagnosis, and/or treatment (e.g., in adult SMA [2]).

We hope that studies such as these, that integrate modern molecular methodologies across cell and tissue models, computational approaches, and clinical studies, will continue to drive progress towards improved neuromuscular health and treatments for these often severe diseases.

## Data Availability

Not applicable.
